# Natural Products from Octocorals of the Genus *Dendronephthya* (Family Nephtheidae)

**DOI:** 10.3390/molecules25245957

**Published:** 2020-12-16

**Authors:** Yung-Husan Chen, Yu-Chia Chang, Yu-Hsin Chen, Li-Guo Zheng, Pin-Chang Huang, Thanh-Hao Huynh, Bo-Rong Peng, You-Ying Chen, Yu-Jen Wu, Lee-Shing Fang, Jui-Hsin Su, Chang-Min Hsu, Ping-Jyun Sung

**Affiliations:** 1 Department of Pharmacy, Xiamen Medical College, Xiamen 361023, Fujian, China; cyxuan@xmmc.edu.cn; 2 Research Center for Chinese Herbal Medicine, Graduate Institute of Healthy Industry Technology, College of Human Ecology, Chang Gung University of Science and Technology, Taoyuan 333324, Taiwan; ycchang03@mail.cgust.edu.tw; 3 National Museum of Marine Biology and Aquarium, Pingtung 944401, Taiwan; kb5634@yahoo.com.tw (Y.-H.C.); t0919928409@gmail.com (L.-G.Z.); foter25632@gmail.com (P.-C.H.); haohuynh0108@gmail.com (T.-H.H.); pengpojung@gmail.com (B.-R.P.); zoeblack0108@gmail.com (Y.-Y.C.); x2219@nmmba.gov.tw (J.-H.S.); 4 Doctoral Degree Program in Marine Biotechnology, National Sun Yat-sen University, Kaohsiung 804201, Taiwan; 5 Department of Marine Biotechnology and Resources, National Sun Yat-sen University, Kaohsiung 804201, Taiwan; lsfang@csu.edu.tw; 6 Doctoral Degree Program in Marine Biotechnology, Academia Sinica, Taipei 115201, Taiwan; 7 Department of Food Science and Nutrition, Meiho University, Pingtung 912009, Taiwan; x00002180@meiho.edu.tw; 8 Center for Environmental Toxin and Emerging-Contaminant Research, Cheng Shiu University, Kaohsiung 833301, Taiwan; 9 Super Micro Mass Research and Technology Center, Cheng Shiu University, Kaohsiung 833301, Taiwan; 10 Graduate Institute of Marine Biology, National Dong Hwa University, Pingtung 944401, Taiwan; 11 Department of Immunology & Rheumatology, Antai Medical Care Corporation Antai Tian-Sheng Memorial Hospital, Pingtung 928004, Taiwan; 12 Chinese Medicine Research and Development Center, China Medical University Hospital, Taichung 404394, Taiwan; 13 Graduate Institute of Natural Products, Kaohsiung Medical University, Kaohsiung 807378, Taiwan

**Keywords:** octocoral, *Dendronephthya*, steroid, natural compound

## Abstract

In this review, 170 natural substances, including steroid, diterpenoid, sesquiterpenoid, peptide, prostaglandin, base, chlorolipid, bicyclolactone, amide, piperazine, polyketide, glycerol, benzoic acid, glycyrrhetyl amino acid, hexitol, pentanoic acid, aminoethyl ester, octadecanone, alkaloid, and a 53-kD allergenic component from octocorals belonging to genus *Dendronephthya*, were listed. Some of these compounds displayed potential bioactivities.

## 1. Introduction

**Figure 1:**
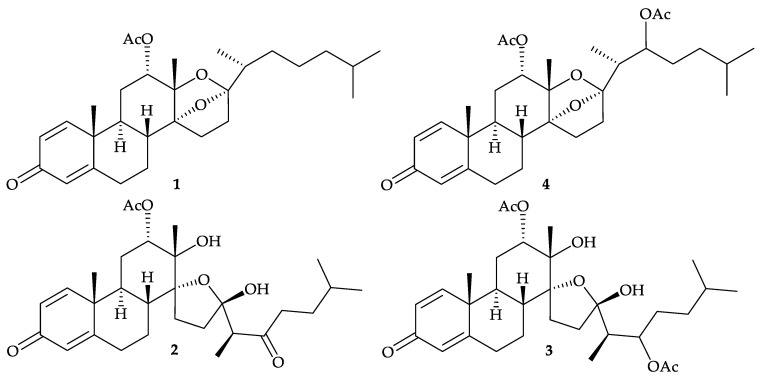
Structures of isogosterones A–D (**1**–**4**).

Octocorals of the genus *Dendronephthya* (phylum Cnidaria, class Anthozoa, subclass Octocorallia, order Alcyonacea, suborder Alcyoniina, family Nephtheidae) [1], distributed in the Indo-Pacific Ocean, have been investigated. Since the initial study in 1999 discovered four antifouling *seco*-steroids, isogosterones A–D (**1**–**4**), from an octocoral *Dendronethphya* sp. collected off the Izu Peninsula, Japan [2] (Figure 1), subsequent studies over the past two decades have yielded a series of interesting secondary metabolites, particularly steroid metabolites. In this article, different types of compounds isolated from *Dendronephthya* spp., were summarized.

## 2. *Dendronephthya gigantea* (Verrill, 1864)

**Figure 2:**
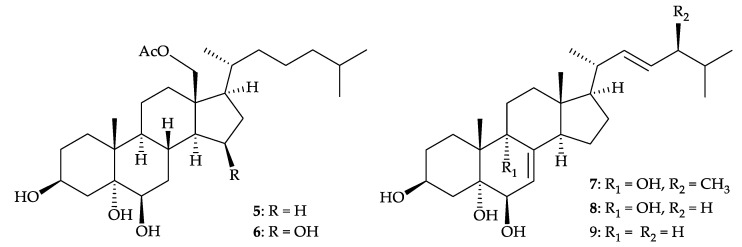
Structures of dendronesterols A (**5**) and B (**6**), (22*E*,24*S*)-24-methyl-cholesta-7,22-diene-3β, 5α,6β,9α-tetrol (**7**), (22*E*)-cholesta-7,22-diene-3β,5α,6β,9α-tetrol (**8**), and (22*E*)-24-norcholesta-7,22- diene-3β,5α,6β-triol (**9**).

The *Dendronephthya* genus includes one common species, *D. gigantea*. Yoshikawa and colleagues isolated five polyhydroxylated sterols, including two new metabolites, dendronesterols A (**5**) and B (**6**), along with three known analogues, (22*E*,24*S*)-24-methyl-cholesta-7,22-diene-3β,5α, 6β,9α-tetrol (**7**) [3], (22*E*)-cholesta-7,22-diene-3β,5α,6β,9α-tetrol (**8**) [3], and (22*E*)-24-norcholesta-7, 22-diene-3β,5α,6β-triol (**9**) [4,5] (Figure 2), from *D. gigantea* collected off the coast of Tokushima, Japan [6]. The study also established the structures of new sterols **5** and **6** by spectroscopic methods. A cytotoxic assay showed that sterol **6** had an IC_50_ value of 5.2 µg/mL in the treatment of L1210 (mouse lymphocytic leukemia) cells [6].

**Figure 3:**
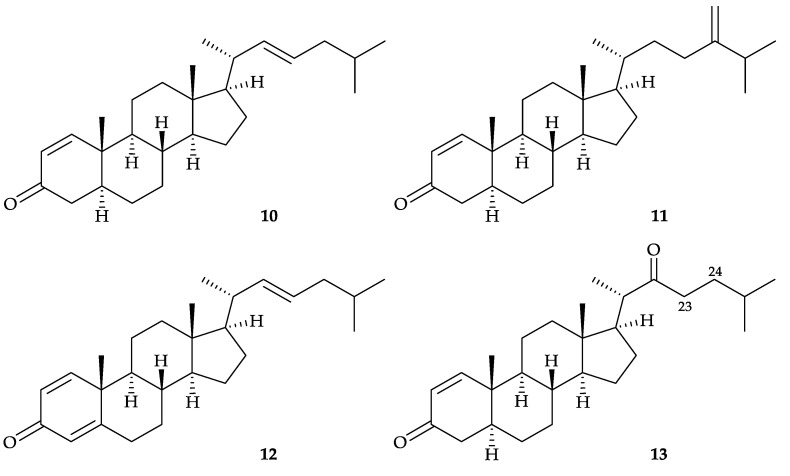
Structures of dendronesterones A–C (**10**–**12**) and cholest-1-ene-3,22-dione (**13**).

In 2004, three new steroids, dendronesterones A–C (**10**–**12**), along with a known steroid, cholest-1-ene-3,22-dione (**13**) [7], were isolated from *D. gigantea*, collected at Green Island, off Taiwan [8] (Figure 3). Structures of steroids **10**–**13** were established by spectroscopic methods, and the ^1^H and ^13^C chemical shifts at C-23 and C-24 in steroid **13** were revised in this study. In the cytotoxic testing, steroids **10** and **13** had ED_50_ values of 9.84 and 8.93 µM, respectively, in the treatment of P-388 (mouse lymphoma) cells, and **13** was cytotoxic toward HT-29 (human colorectal adenocarcinoma) cells with an ED_50_ value of 9.03 µM [8]. 

**Figure 4:**
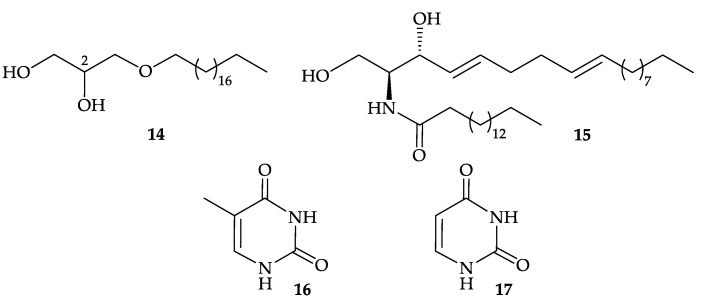
Structures of (±)-1-nonadecyloxy-2,3-propanediol (**14**) and (2*S*,3*R*,4*E*,8*E*)-*N*-hexadecanoyl- 2-amino-4,8-octadecadiene-1,3-diol (**15**), thymine (**16**), and uracil (**17**).

Furthermore, two known metabolites, including a monoalkyl glycerol ether (±)-1-non- adecyloxy-2,3-propanediol (**14**) [9], a ceramide, (2*S*,3*R*,4*E*,8*E*)-*N*-hexadecanoyl-2-amino-4,8- octadecadiene-1,3-diol (**15**) [10,11,12,13,14], as well as two bases, thymine (**16**) and uracil (**17**), (Figure 4), were isolated from the organic extract of *D. gigantea*, collected in the area of Jeju Island, Korea [15]. The structures of metabolites **14**–**17** were established by spectroscopic methods and by comparison of their physical and spectral data with those of literature values and glycero **14** was found to be cytotoxic toward A549 (human lung epithelial carcinoma), HT-29, HT-1080 (human connective tissue epithelial fibrosarcoma), and SNU-638 (human gastric adenocarcinoma) cells with IC_50_ values of 15.1, 14.5, 13.7, and 15.5 µg/mL, respectively [15]. Glycerol **14** was not optically active (${\left[\alpha \right]}_{D}^{25}$ 0.00 (*c* 0.134, MeOH)), indicating that this compound is a racemic mixture. Thus, the stereogenic center C-2 in **14** was not determined [15]. Sphingolipid **15** showed cytotoxicity against human peripheral blood mononuclear cells (PBMC) with an ED_50_ of 20 µg/mL [13].

**Figure 5:**
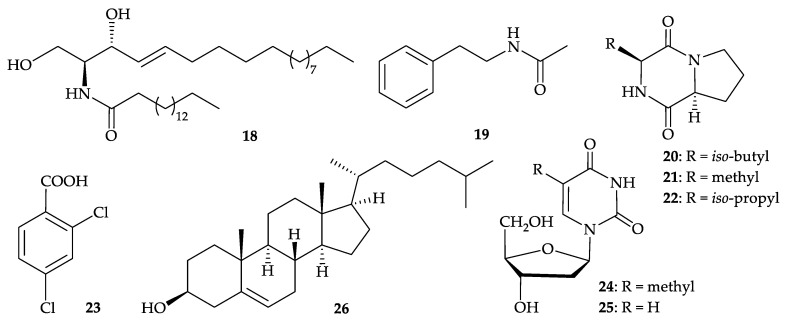
Structures of (2*S*,3*R*,4*E*)-*N*-hexadecanoyl-2-amino-4-octadecane-1,3-diol (**18**), *N*-phenethyl -acetamide (**19**), cyclo-(Leu-Pro) (**20**), cyclo-(Ala-Pro) (**21**), cyclo-(Val-Pro) (**22**), 2,4-dichlorobenzonic acid (**23**), thymidine (**24**), 2′-deoxyuride (**25**), and cholesterol (**26**).

Eight well known secondary metabolites, including (2*S*,3*R*,4*E*,8*E*)-*N*-hexadecanoyl-2-amino-4,8- octadecadiene-1,3-diol (**15**) [10,11,12,13,14] (Figure 4), (2*S*,3*R*,4*E*)-*N*-hexadecanoyl-2-amino-4-octadecane-1,3- diol (**18**) [10,16], *N*-phenethylacetamide (**19**) [17,18,19,20,21], cyclo-(Leu-Pro) (**20**), cyclo-(Ala-Pro) (**21**), cyclo- (Val-Pro) (**22**) [22], 2,4-dichlorobenzonic acid (**23**) [23], thymidine (**24**) [24,25,26,27,28,29,30,31,32], 2′-deoxyuridine (**25**) [27,28,29,30,32,33], and cholesterol (**26**) [30] (Figure 5), were isolated from *D. gigantea*, collected from the South China Sea [34]. The structures of compounds **15** and **18**–**26** were elucidated by spectral data and by comparison with the spectral and physical data of other known compounds [34].

**Figure 6:**
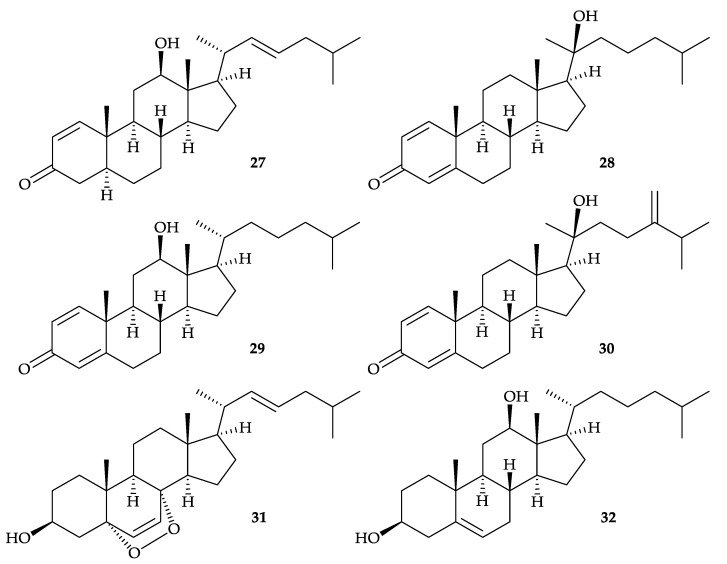
Structures of 3-oxocholest-1,22-dien-12β-ol (**27**) 3-oxocholest-1,4-dien-20β-ol (**28**), 3-oxo- cholest-1,4-dien-12β-ol (**29**), (20*S*)-20-hydroxyergosta-1,4,24-trien-3-one (**30**), 5α,8α-epidioxy- cholesta-6,22-dien-3β-ol (**31**), and 5-cholestene-3β,12β-diol (**32**).

In 2012, six steroids, including three new compounds, 3-oxocholest-1,22-dien-12β-ol (**27**), 3- oxocholest-1,4-dien-20β-ol (**28**), 3-oxocholest-1,4-dien-12β-ol (**29**), along with three known analogues, (20*S*)-20-hydroxyergosta-1,4,24-trien-3-one (**30**) [35], 5α,8α-epidioxycholesta-6,22-dien-3β-ol (**31**) [36], and 5-cholestene-3β,12β-diol (**32**) [37] (Figure 6), were isolated from *D. gigantea*, collected near Geo-Je Island, South Korea [38]. The structures for steroids **27**–**32** were established by spectroscopic methods. Steroids **27**–**31** displayed inhibitory activity against farnesoid X-activated receptor (FXR) with IC_50′_s 14, 15, 100, 22, and 61 µM, respectively, and were not cytotoxic toward the CV-1 cells (*Cercopithecus aethiops*, African green monkey kidney cells) [38].

**Figure 7:**
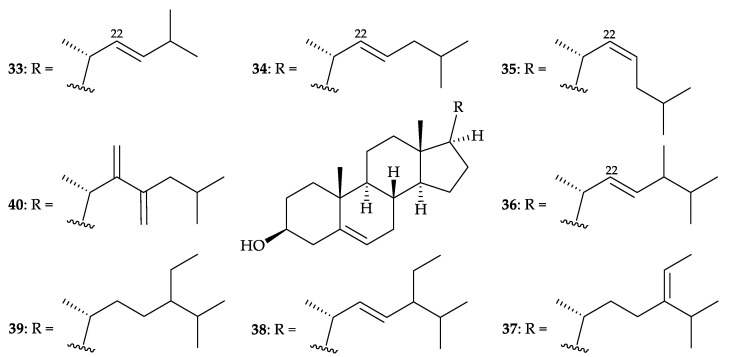
Structures of 26,27-dinorergosta-5,22-dien-3β-ol (**33**), cholesta-5,22-dien-3β-ol (including 22-*trans* form **34** and 22-*cis* form **35**), ergosta-5,22-dien-3β-ol (**36**), stigmasta-5,24-dien-3β-ol (= fucosterol) (**37**), stigmasta-5,22-dien-3β-ol (**38**), stigmasta-5-en-3β-ol (**39**), and 22,23-methylene- cholesterol (**40**).

In 2017, Jeon and Lee’s group reported the isolation of a mixture consisting nine 3β-hydroxy- Δ^5^-steroidal congeners, including 26,27-dinorergosta-5,22-dien-3β-ol (**33**) [39], cholesta-5,22-dien- 3β-ol (including 22-*trans* form **34** and 22-*cis* form **35**) [40], cholest-5-en-3β-ol (= cholesterol) (**26**) [30] (Figure 5), ergosta-5,22-dien-3β-ol (**36**) [41], stigmasta-5,24-dien-3β-ol (= fucosterol) (**37**) [42,43,44,45,46,47,48], stigmasta-5,22-dien-3β-ol (**38**) [48], stigmasta-5-en-3β-ol (**39**) [48], and 22,23-methylenecholesterol (**40**) [49] (Figure 7), from *D. gigantea* collected from Jeju Island, South Korea [50]. The structures for all sterols **26** and **33**–**40** were determined by GC-MS/MS analysis. In lipopolysaccharides (LPS)- stimulated RAW cells, this mixture inhibited nitric oxide (NO) and prostaglandin E_2_ (PGE_2_) production via the downregulation of inducible nitric oxide synthase (iNOS) and cyclooxygenase-2 (COX-2) inflammatory mediators. This sterol-rich mixture also suppressed the expression of proinflammatory cytokines, including tumor necrosis factor-α (TNF-α), interleukin 1β (IL-1β), and interleukin 6 (IL-6). The anti-inflammatory effects of this sterol-rich mixture was confirmed in an LPS-stimulated in vivo zebrafish model by the downregulation of iNOS and COX-2 expression, inhibition of NO and reactive oxygen species (ROS) levels, and increased cytoprotective effects against LPS-induced toxicity [50]. Furthermore, this sterol-rich fraction was found to exhibit cytotoxicity toward HL-60 (human acute promyelocytic leukemia) and MCF-7 (Michigan Cancer Foundation-7, human invasive ductal carcinoma) cells with IC_50_ values of 13.59 and 29.41 µg/mL [51], and one of the mixtures, stigmasta-5-en-3β-ol (**39**), displayed cytotoxicity on HL-60 and MCF-7 cells with IC_50_ values of 37.82 and 45.17 µg/mL, respectively [52].

**Figure 8:**
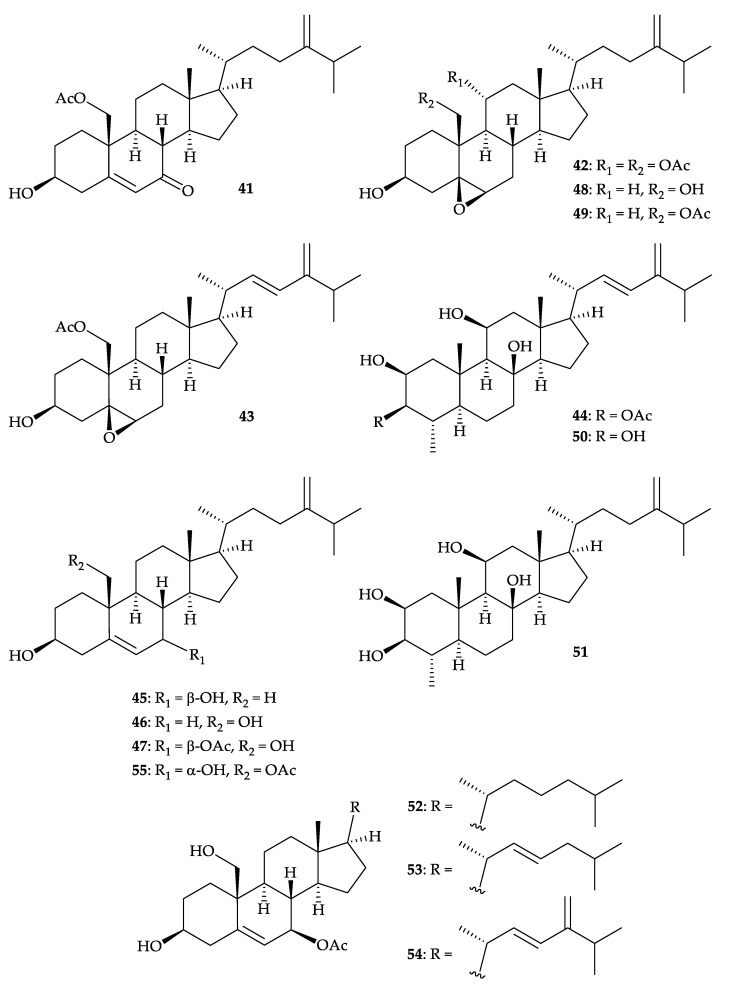
Structures of 7-dehydroerectasteroid F (**41**), 11α-acetoxyarmatinol A (**42**), 22,23-di- dehydroarmatinol A (**43**), 3-*O*-acetylhyrtiosterol (**44**), 24-methylene-5-cholesten-3β,7β-diol (**45**), 24- methylene-5-cholesten-3β,19-diol (**46**), 24-methylene-5-cholesten-3β,19-diol-7β-monoacetate (**47**), 5,6-epoxylitosterol (**48**), armatinol A (**49**), hyrtiosterol (**50**), (2β,3β,4α,5α,8β,11β)-4-methylergost-24 (28)-ene-2,3,8,11-tetrol (**51**), and erectasteroids C–F (**52**–**55**).

Fifteen steroids, including four new compounds, 7-dehydroerectasteroid F (**41**), 11α- acetoxyarmatinol A (**42**), 22,23-didehydroarmatinol A (**43**), and 3-*O*-acetylhyrtiosterol (**44**), as well as 11 known steroids, 24-methylene-5-cholesten-3β,7β-diol (**45**) [53], 24-methylene-5-cholesten-3β, 19-diol (= litosterol) (**46**) [54], 24-methylene-5-cholesten-3β,19-diol-7β-monoacetate (**47**) [55], 5,6- epoxylitosterol (**48**) [54], armatinol A (**49**) [56], hyrtiosterol (**50**) [57,58], (2β,3β,4α,5α,8β,11β)-4- methylergost-24-ene-2,3,8,11-tetrol (**51**) [58], and erectasteroids C–F (**52**–**55**) [59] (Figure 8), were isolated from *D. gigantea*, collected from the inner coral reef of Meishan, Hainan Province, China [60]. The structures of new steroids **41**–**43** were elucidated by comprehensive spectroscopic analysis and steroid **41** was found to show protection against hydrogen-peroxide (H_2_O_2_)-induced oxidative damage in neuron-like PC-12 (rat adrenal gland pheochromocytoma) cells by promoting nuclear translocation of nuclear factor erythroid 2-related factor 2 (Nrf2) and enhancing the expression of heme oxygenase-1 (HO-1) [60]. 

## 3. *Dendronephthya griffini* (Roxas, 1933)

**Figure 9:**
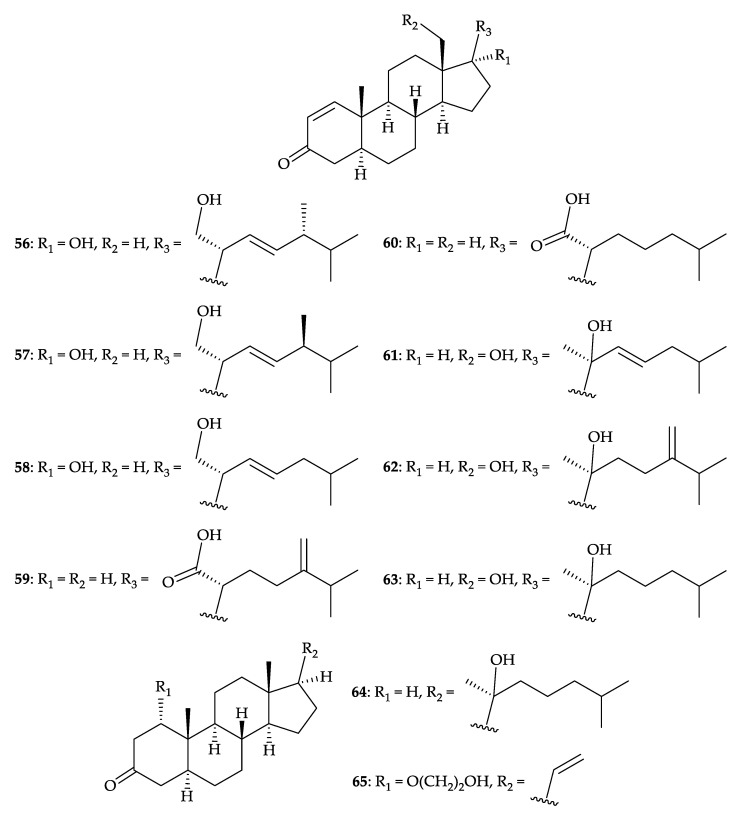
Structures of griffinisterones A–I (**56**–**64**) and griffinipregnone (**65**).

Ten new steroids, griffinisterones A–I (**56**–**64**) and griffinipregnone (**65**) (Figure 9), were obtained from *D. griffini* specimens collected by a bottom trawl net at depths from 200 to 100 m at Taiwan Straight in December 2004 [61,62]. The structures of steroids **56**–**65** were determined by spectroscopic methods and the configuration of griffinisterone A (**56**) was further confirmed by a single-crystal X-ray diffraction analysis [61,62]. The absolute stereochemistry of griffinisterone E (**60**) was determined by the application of a modified phenylglycine methyl ester (PGME) method [61]. Anti-inflammatory assays revealed that griffinisterones A–D (**56**–**59**), F–H (**61**–**63**), and griffinipregnone (**65**), reduced the levels of iNOS protein to 49.7, 48.9, 8.1, 29.8, 13.4, 6.5, 15.4, and 59.6%, respectively, at a concentration of 10 µM [61,62]. At the same concentration, griffinisterones F (**61**), G (**62**), and griffinipregnone (**65**), reduced the levels of COX-2 protein to 61.7, 31.5, and 52.3%, respectively [62].

**Figure 10:**
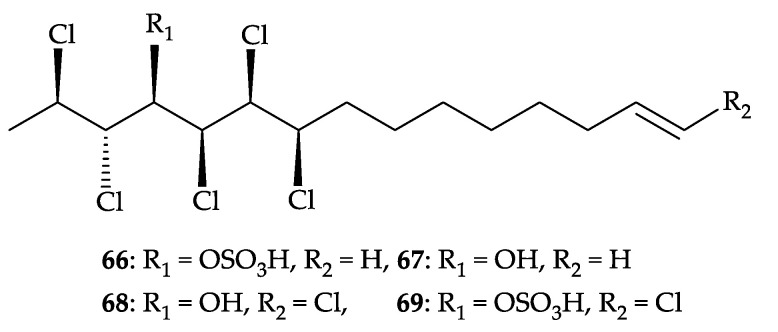
Structures of polychlorolipids **66**–**69**.

Furthermore, two new interesting polychlorolipids, (2*R*,3*S*,4*R*,5*S*,6*S*,7*R*)-2,3,5,6,7-pentachloro- pentadec-14-en-4-yl hydrogen sulfate (**66**), (2*R*,3*S*,4*R*,5*S*,6*S*,7*R*)-2,3,5,6,7-pentachloropentadec-14- en-4-ol (**67**), and a new natural substance, (2*R*,3*S*,4*R*,5*S*,6*S*,7*R*,*E*)-2,3,5,6,7,15-hexachloropentadec- 14-en-4-ol (**68**) [63,64], along with a known analogue, chlorosulfolipid (**69**) [63,64] (Figure 10), were obtained from *D. griffini* [65]. The structures of chlorolipids **66**–**69** were determined by extensive spectroscopic analysis and by comparison of the NMR data with those of known compounds. It was found that chlorolipid **68** has been prepared from the hydrolysis of **69** [63] and by a total synthesis of racemic **68** [64]. Chlorolipid **68** was isolated for the first time from a natural source and the compounds of this type was isolated for the first time from the soft corals [65].

## 4. *Dendronephthya hemprichi* (Klunzinger, 1877)

**Figure 11:**
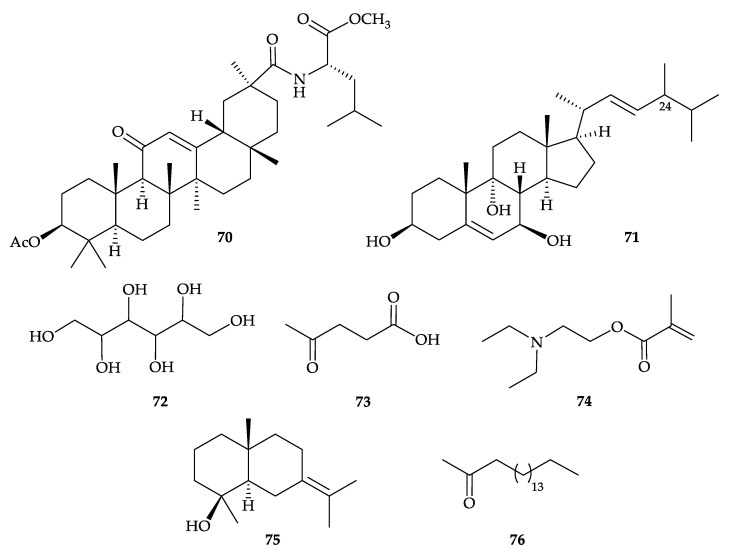
Structures of dendrophen (**70**), dendrotriol (**71**), hexiol (**72**), 4-oxo-pentanoic acid (**73**), 2- methyl-acrylic acid 2-diethylaminoethyl ester (**74**), juniper camphor (**75**), and 2-octadecanone (**76**).

Chemical investigation of the extract of *D. hemprichi*, collected from the Red Sea, Egypt, delivered a novel glycyrrhetyl amino acid, dendrophen (**70**), a new sterol, dendrotriol (**71**), along with the well-known metabolites, cholesterol (**26**) [30] (Figure 4) and hexitol (**72**) [66]. The structures of new compounds **70** and **71** were established by spectroscopic methods, although the stereocehmsitry for C-24 stereogenic center in **71** was not determined [66]. Furthermore, chromatography separation of the low-polarity components of *D. hemprichi* extract afforded 4-oxo- pentanoic acid (**73**), 2-methyl-acrylic acid 2-diethylaminoethyl ester (**74**), juniper camphor (**75**), and 2-octadecanone (**76**) (Figure 11) [66].

## 5. *Dendronephthya mucronata* (Pütter, 1900)

**Figure 12:**
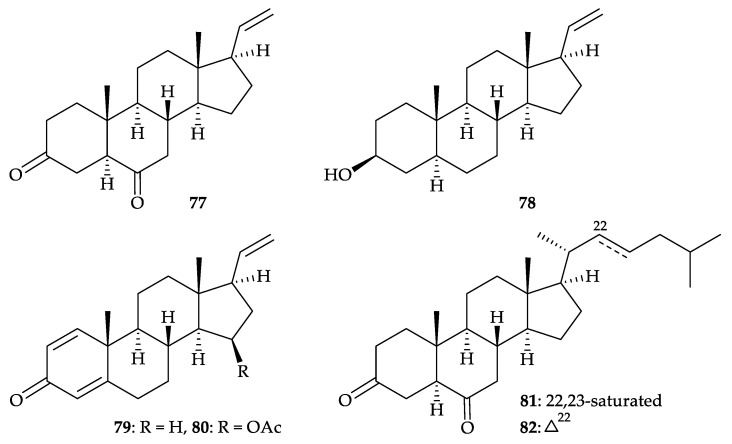
Structures of 5α-pregn-20-en-3,6-dione (**77**), 5α-pregn-20-en-3β-ol (**78**), 1,4,20-pregna- trien-3-one (**79**), 15β-acetoxypregna-1,4,20-trien-3-one (**80**), 5α-cholestan-3,6-dione (**81**), and 5α- cholest-22-en-3,6-dione (**82**).

A new pregane-type steroid 5α-pregn-20-en-3,6-dione (**77**), along with five known steroids, 5α- pregn-20-en-3β-ol (**78**) [67,68,69], 1,4,20-pregnatrien-3-one (**79**) [70,71,72,73,74], 15β-acetoxypregna-1,4,20-trien-3-one (**80**) [73,75], 5α-cholestan-3,6-dione (**81**) [76,77,78], and 5α-cholest-22-en-3,6-dione (**82**) [79], (Figure 12), were isolated from *D. mucronate* collected from waters off Phu Quoc Islands, Kien Giang, Vietnam in 2018 [80]. The structure of new steroid **77** was elucidated by spectroscopic method. Steroids **78** and **81** showed moderate inhibitory effects on LPS-induced NO formation in RAW264.7 murine macrophage cells with IC_50_ values of 30.15 and 35.97 µM, respectively.

**Figure 13:**
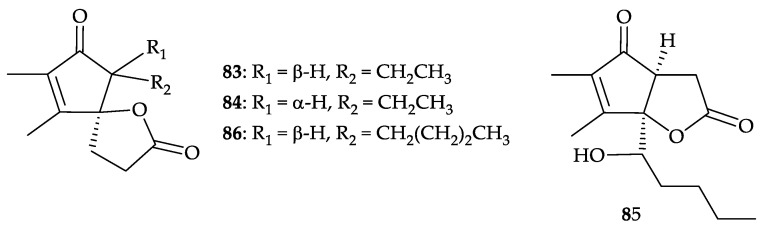
Structures of dendronephthyones A–C (**83**–**85**) and suberosanone B (**86**).

Furthermore, three new bicyclo lactones, dendronephthyones A–C (**83**–**85**), along with a known analogue, suberosanone B (**86**) [81] (Figure 13), were isolated from the methanol extract of the same target material *D. mucronata* [82]. Structures of lactones **83**–**86** were established by spectroscopic methods and these four compounds exhibited cytotoxicity toward HeLa (human papillomavirus-related endocervical adenocarcinoma) cells with IC_50_ values of 32.48, 30.12, 35.45, and 14.45 µM, respectively [82].

## 6. *Dendronephthya nipponica* (Utinomi, 1952)

A red soft-coral *D. nipponica* cause spiny lobster fisherman living along the coast of Miyazaki Prefecture, Japan to develop occupational allergies. In order to understand the allergic mechanism, a new 53-kD allergenic component (Den n 1) (**87**) was purified and the *N*-terminal amino of this allergen component was determined and identified as Asp-Asp-IIe-Asn-Arg-Tyr-Ala-Phe-Asp-Asn-Lys-IIe-Asn-Asp-Lys-Leu-Phe-Asp-His-Trp-Gln-Ser [83].

## 7. *Dendronephthya puetteri* (Kükenthal, 1905)

**Figure 14:**
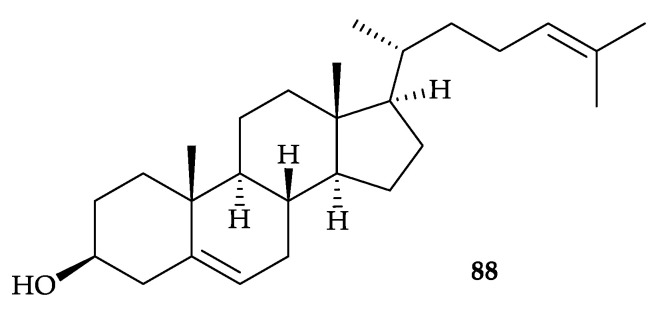
Structure of cholesta-5,24-dien-3β-ol (**88**).

In 2018, Jeon’s group reported the isolation of a 3β-hydroxy-Δ^5^-steroidal congener, consisting of six sterols, cholesterol (**26**) [30] (Figure 4), cholesta-5,22-dien-3β-ol (**34**) [40], ergosta-5,22-dien-3β-ol (**36**) [41], stigmasta-5-en-3β-ol (**39**) [48], 22,23-methylenecholesterol (**40**) [49] (Figure 7), and cholesta-5,24-dien-3β-ol (**88**) [84] (Figure 14), from *D. puetteri*, collected from the Jeju Island, South Korea [85]. The structures for all sterols **26**, **34**, **36**, **39**, **40**, and **88** were determined by GC-MS/MS analysis [85]. In lipopolysaccharides (LPS)-stimulated RAW264.7 cells, this mixture inhibited nitric oxide (NO) production with an IC_50_ value of 6.54 µg/mL. Moreover, this congener reduced the level of PGE_2_ TNF-α, IL-1β, and IL-6. The anti-inflammatory effects of this sterol-rich mixture was confirmed in an LPS-stimulated in vivo zebrafish model by the downregulation of NO, iNOS, COX-2, ROS production and cell death [85,86], and this sterol rich congener showed cytotoxicity toward HL-60 and MCF-7 cells with IC_50_ values of 25.27 and 22.81 µg/mL, respectively [87].

## 8. *Dendronephthya rubeola* (Henderson, 1909)

**Figure 15:**
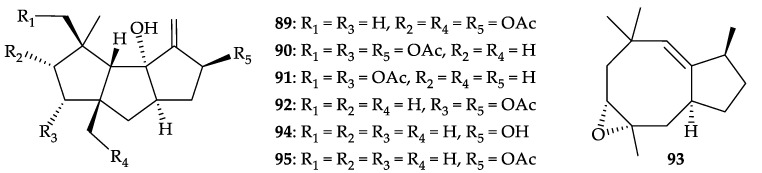
Structures of 2α,8β,13-triacetoxycapnell-9-ene-10α-ol (**89**), 3α,8β,14-triacetoxy- capnell-9-ene-10α-ol (**90**), 3α,14-diacetoxycapnell-9-ene-8β,10α-diol (**91**), 3α,8β-diacetoxy- capnell-9-ene-10α-ol (**92**), 3α,4α-epoxyprecapnell-10-ene (**93**), capnell-9-ene-8β,10α-diol (**94**), and 8β-acetoxycapnell-9-ene-10α-ol (**95**).

Four new acetoxycapnellenes, 2α,8β,13-triacetoxycapnell-9-ene-10α-ol (**89**), 3α,8β,14-tri- acetoxycapnell-9-ene-10α-ol (**90**), 3α,14-diacetoxycapnell-9-ene-8β,10α-diol (**91**), 3α,8β-di- acetoxycapnell-9-ene-10α-ol (**92**), and the first epoxyprecapnellene, 3α,4α-epoxyprecapnell-10- ene (**93**), as well as two known analogues, capnell-9-ene-8β,10α-diol (**94**) [88,89] and 8β-acetoxy- capnell-9-ene-10α-ol (**95**) [88,90] (Figure 15), were obtained from *D. rubeola*, collected from the waters near Bali, Indonesia [91]. Structures of **89**–**95** were established by spectroscopic methods. Compounds **94** and **95** displayed antiproliferative activity against L-929 (murine connective tissue fibroblasts) (GI_50_ = 6.8, 20.9 µM) [91]; **94** displayed cytotoxicity toward HL-60, K-562 (human chronic myelogenous leukemia), G-402 (human renal leiomyoblastoma), MCF-7, HT-115 (human colon carcinoma), and A-2780 (human ovarian endometrioid adenocarcinoma) cells with IC_50_ values of 51, 0.7, 42–51, 93, 63, and 9.7 µM, respectively [89]. Compounds **94** and **95** also showed cytotoxicity toward HeLa cells (CC_50_ = 7.6, 9.4 µM) [91]. It is interesting to note that compound **94** (capnell-9-ene-8β,10α-diol) inhibited the interaction of oncogenic transcription factor Myc (a family of regulator genes and proto-oncogenes that code for transcription factors) with its partner protein Max (inhibition = 77%) in yeast [91]. 

## 9. *Dendronephthya studeri* (Ridley, 1884)

**Figure 16:**
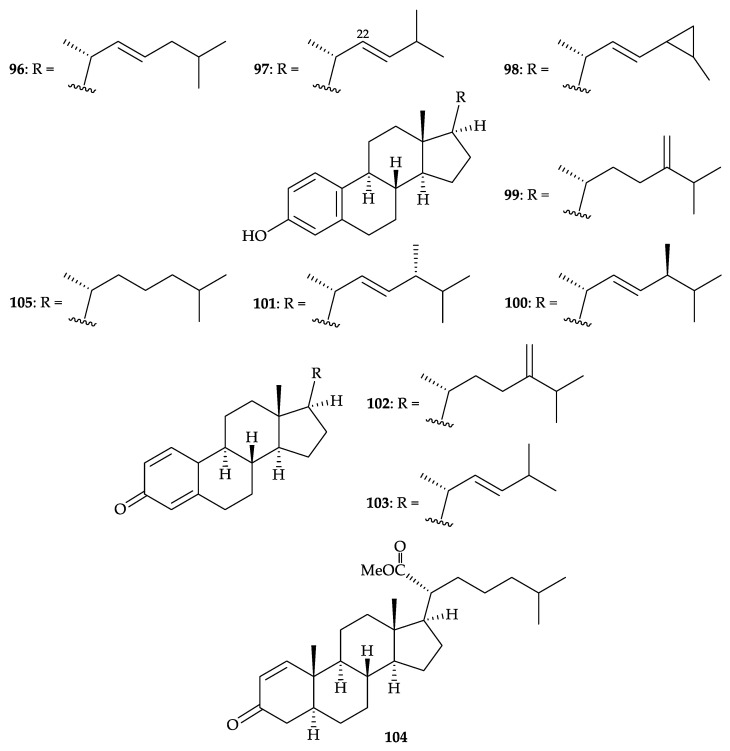
Structures of (22*E*)-19-norcholesta-1,3,5,22-tetraen-3-ol (**96**), (22*E*)-19,24-dinor- cholesta-1,3,5,22-tetraen-3-ol (**97**), (22*E*)-24,26-cyclo-19-norcholesta-1,3,5,22-tetraen-3-ol (**98**), 24-methylene-19-norcholesta-1,3,5,22-tetraen-3-ol (**99**), (22*E*,24*S*)-24-methyl-19-norcholesta-1,3,5 (10),22-tetraen-3-ol (**100**), (22*E*,24*R*)-24-methyl-19-norcholesta-1,3,5,22-tetraen-3-ol (**101**), 24- methylenecholesta-1,4,22-trien-3-one (**102**), (22*E*)-24-cholesta-1,4,22-trien-3-one (**103**), methyl spongoate (**104**), and 19-norcholesta-1,3,5-trien-3-ol (**105**).

Eleven steroids, including eight new metabolites, (22*E*)-19-norcholesta-1,3,5,22-tetraen-3-ol (**96**), (22*E*)-19,24-dinorcholesta-1,3,5,22-tetraen-3-ol (**97**), (22*E*)-24,26-cyclo-19-norcholesta-1,3,5 (10),22-tetraen-3-ol (**98**), 24-methylene-19-norcholesta-1,3,5,22-tetraen-3-ol (**99**), (22*E*,24*S*)-24- methyl-19-norcholesta-1,3,5,22-tetraen-3-ol (**100**), (22*E*,24*R*)-24-methyl-19-norcholesta-1,3,5, 22-tetraen-3-ol (**101**), 24-methylenecholesta-1,4,22-trien-3-one (**102**), and (22*E*)-24-cholesta-1,4,22- trien-3-one (**103**), which all were found to be characterized by either the presence of an aromatic ring or a cross-conjugated dienone system in ring A, as well as three known steroids, methyl spongoate (**104**) [92], 19-norcholesta-1,3,5-trien-3-ol (**105**) [93,94], and dendronesterone C (**12**) (Figure 3) [8], were obtained from *D. studeri*, collected off the coast of Xiaodong Sea, Hainan Province, China [95] (Figure 16). Structures of isolates **12** and **96**–**105** were established by spectroscopic analysis and by comparison of their NMR data with those reported in the literature. Steroid **104** exhibited cytotoxicity against BEL-7402 (human papillomavirus-related endocervical adenocarcinoma), A-549, HT-29, and P-388 cells with IC_50_ values of 0.14, 5, 5, and 3.8 µg/mL [92]. 

## 10. *Dendronephthya* spp.

*Dendronephthya* is a genus of octocoral belonging to the family Nephtheidae and there are over 250 described species in this genus. In 1990, Katrich and colleagues identified the correlation between the number of particular phospholipids (PhLs) and prostaglandins (PGs) that influenced the prostaglandin-like activities of the extracts from (1) *Dendronephthya* sp., collected in the region of the Great Barrier Reef, Australia and (2) *Dendronephthya* sp., collected in Vietnam [96].

**Figure 17:**
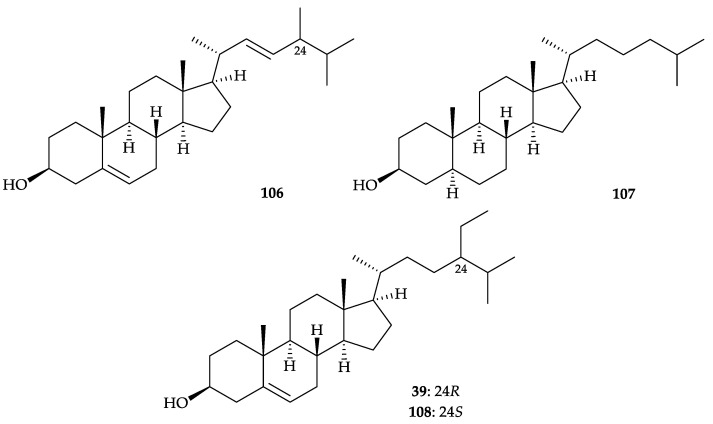
Structures of pincsterol (24*S*), brassicasterol (24*R*) (**106**), β-cholestanol (**107**), β-sitosterol (**39**), and clionasterol (**108**).

An acetone extract from *Dendronephthya* sp., collected in 1990, off the Chichi-jima and Haha-jima Islands in the Ogasawara Islands, Japan, showed a high level of antifouling activity against the blue mussel *Mytilus edulis* [97]. Purification of the extract gave mixtures of sterols and fatty acids as active components. In the sterol mixture, there are several sterols, (24*S*)-24-methylcholesta-5(*E*),22- dien-3β-ol (= pincsterol) or (24*R*)-24-methylcholesta-5(*E*),22-dien-3β-ol (= brassicasterol) (**106**) [98], cholesterol (**26**) [30] (Figure 5), β-sitosterol (stigmasta-5-en-3β-ol) (**39**) [48], and β-cholestanol (5α- cholestan-3β-ol) (**107**) [99] were identified and sterol **39** in this study [97] was found to contain 35% of a 24*S* epimer (clionasterol) (**108**) [100,101] (Figure 17). Sterol **39** had the highest antifouling activity among sterols **26**, **39**, and **107 [97]**. Moreover, a fatty acid mixture, showing the presence of saturated and unsaturated fatty acids with a chain length of C_12_ to C_22_, being rich in C_16_ and C_18_ acids as active constituents in antifouling activity [97].

**Figure 18:**
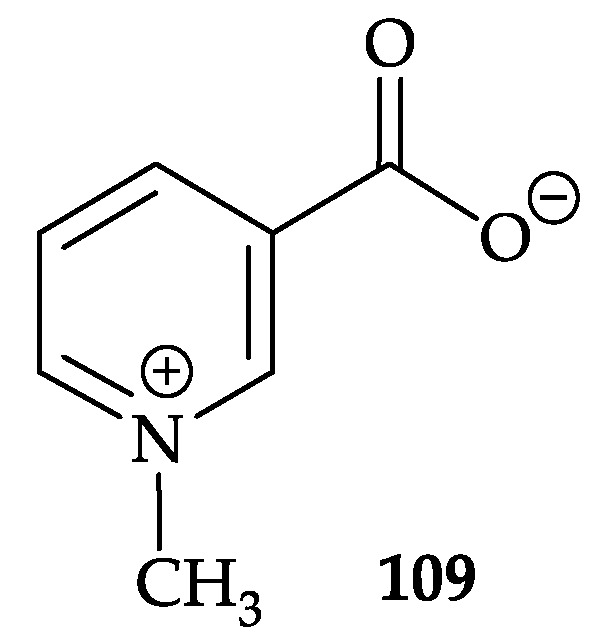
Structure of trigonelline (**109**).

Kawamata et al. isolated an antifouling substance, trigonelline (**109**) (Figure 18), from *Dendronephthya* sp. collected at Chichijima Island in the Ogasawara Islands [102]. The structure of **109** was elucidated by spectroscopic methods and this compound showed the same level of settling-inhibitory activity against the acorn barnacle *Balanus amphitrite* larvae as CuSO_4_ [102,103].

In 1999, the ethanol extract of two soft coral specimens *Dendronephthya* (Roxasia) sp. and *Dendronephthya* (Morchellana) sp., collected off the Gopalpur coast, Bay of Bengal, were found to display attachment inhibitory activity against the settlement of cyprids of barnacle *Balanus amphitrite* [104], and the extract was claimed to contain natural non-toxic antifouling agents, although no natural products was reported to be active components.

Research by a group in Japan identified four new antifouling *seco*-steroids, isogosterones A–D (**1**–**4**) (Figure 1) from an octocoral identified as *Dendronethphya* sp. collected off the Izu Peninsula, Japan [2], and their structures were elucidated on the basis of spectroscopic data. This is the first time to isolate naturally occurring 13,17-secosteroids. It is interesting to note that secosteroids **3** and **4** were interconvertible in CHCl_3_ and **3** was detected as the hydrolyzed product of **4 [2]**. These four secosteroids displayed activity to inhibit the settlement of *B. amphitrite* cyprid larvae with an EC_50_ values of 2.2 µg/mL

**Figure 19:**
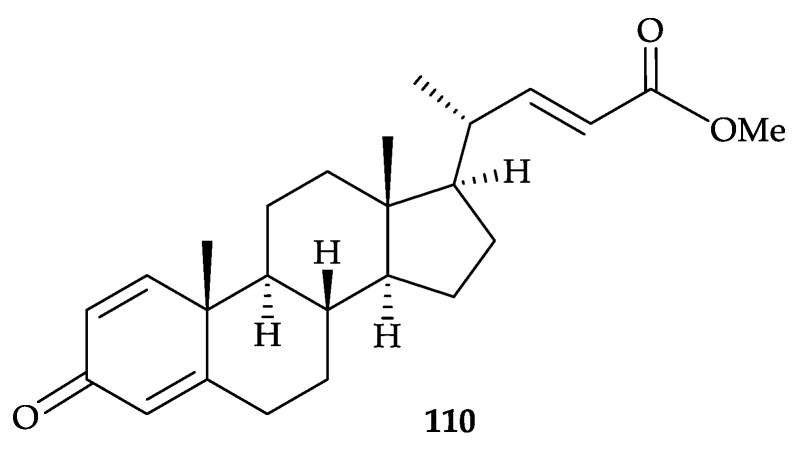
Structure of methyl 3-oxochola-4,22-dien-24-oate (**110**).

Furthermore, a new steroid, methyl 3-oxochola-4,22-dien-24-oate (**110**) (Figure 19) [105], from *Dendronephthya* sp. collected off the Kii Peninsula, Japan, and determined its structure using spectroscopic methods [105]. Steroid **110** was lethal to cyprids of *B. amphitrite* at 100 µg/mL (LD_100_) but did not inhibit larval settlement of *B. amphitrite* [105].

**Figure 20:**
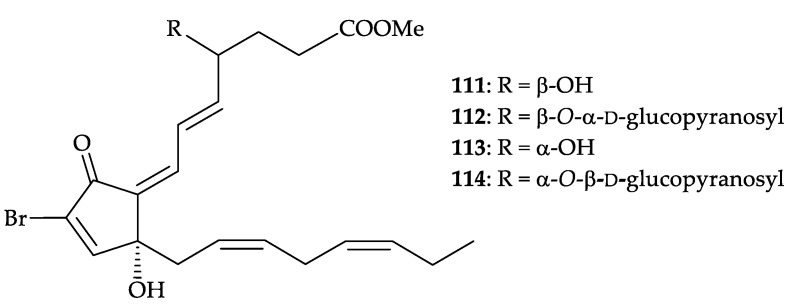
Structures of oxylipins **112**–**114**.

Four new brominated oxylipins, (4*S*,5*E*,7*Z*,12*R*,14*Z*,17*Z*)-4-hydroxy-17,18-didehydrobromo- vulone-3 (**111**), (4*S*,5*E*,7*Z*,12*R*,14*Z*,17*Z*)-4-(α-d-glucopyranosyloxy)-17,18-didehydrobromovulone-3 (**112**), (4*R*,5*E*,7*Z*,12*R*,14*Z*,17*Z*)-4-hydroxy-17,18-didehydrobromovulone-3 (**113**), and (4*R*,5*E*,7*Z*,12*R*, 14*Z*,17*Z*)-4-(β-D-glucopyranosyloxy)-17,18-didehydrobromovulone-3 (**114**), (Figure 20) were isolated from *Dendronephthya* spp. (red variety—for compounds **111** and **112**; yellow variety—for compounds **113** and **114**) collected in the Gulf of Aqaba in the Red Sea (Eilat, Israel) [106]. The structures, including the absolute configurations of oxylipins **111**–**114**, were determined by spectroscopic and chemical methods. All the isolates showed significant inhibition of the growth of crown gall tumors on potato disks inoculated with *Agrobacterium tumefaciens* and gave positive responses in a brine shrimp toxicity toward *Artemia salina*; these compounds showed antibacterial activity against the Gram-(+) bacteria *Staphylococcus aureus* and *Bacillus subtilis* [106].

**Figure 21:**
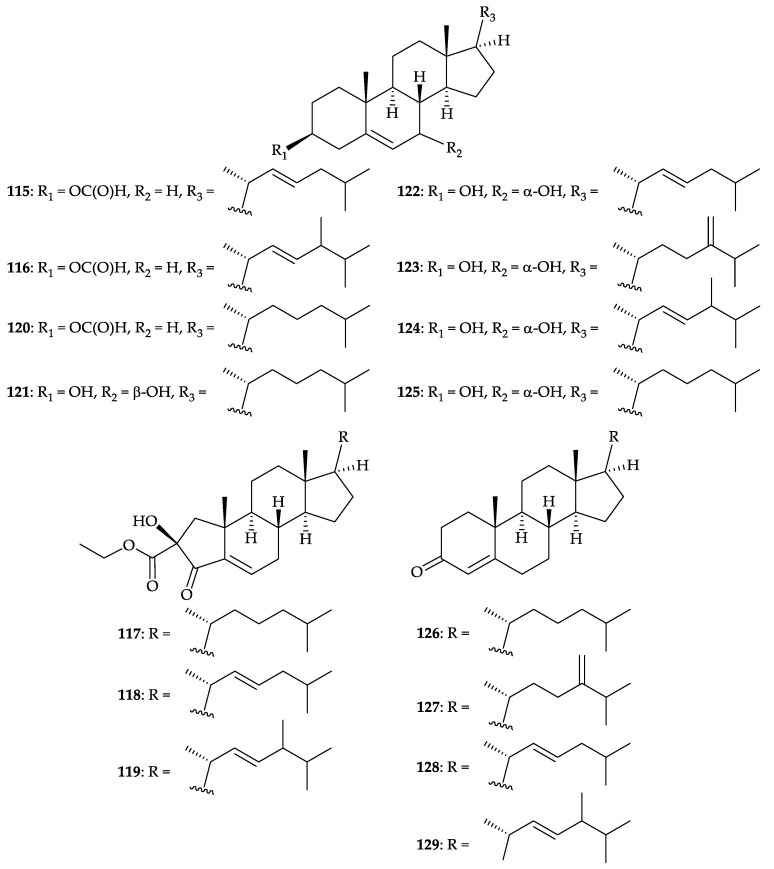
Structures of (22*E*)-3-*O*-β-formylcholest-5,22-diene (**115**), (22*E*)-3-*O*-β-formyl-24-methyl- cholest-5,22-diene (**116**), 2-ethoxycarbonyl-2-β-hydroxy-A-nor-cholest-5-ene-4-one (**117**), (22*E*)-2- ethoxycarbonyl-2-β-hydroxy-A-nor-cholest-5,22-diene-4-one (**118**), (22*E*)-2-ethoxycarbonyl-2-β- hydroxy-24-methyl-A-nor-cholest-5,22-diene-4-one (**119**), 3-β-formyloxycholest-5-ene (**120**), 3β,7β- dihydroxycholest-5-ene (**121**), (22*E*)-3β,7α-dihydroxy-cholest-5,22-diene (**122**), 3β,7α-dihydroxy-24- methylene-cholest-5-ene (**123**), 3β,7α-dihydroxy-24-methyl-cholest-5,22-diene (**124**), 3β,7α- dihydroxy-cholest-5-ene (**125**), cholest-4-ene-3-one (**126**), 24-methylene-cholest-4-ene-3-one (**127**), (22*E*)-cholest-4,22-dien-3-one (**128**), and (22*E*)-24-methyl-cholest-4,22-dien-3-one (**129**).

Fifteen steroids, including five new compounds, (22*E*)-3-*O*-β-formylcholest-5,22-diene (**115**), (22*E*)-3-*O*-β-formyl-24-methyl-cholest-5,22-diene (**116**), 2-ethoxycarbonyl-2-β-hydroxy-A-nor- cholest-5-ene-4-one (**117**), (22*E*)-2-ethoxycarbonyl-2-β-hydroxy-A-nor-cholest-5,22-diene-4-one (**118**), (22*E*)-2-ethoxycarbonyl-2-β-hydroxy-24-methyl-A-nor-cholest-5,22-diene-4-one (**119**), a new natural steroid, 3-β-formyloxycholest-5-ene (**120**) [107], as well as nine known steroids, 3β,7β-dihydroxy- cholest-5-ene (**121**) [108,109], (22*E*)-3β,7α-dihydroxy-cholest-5,22-diene (**122**) [110], 3β,7α- dihydroxy-24-methylene-cholest-5-ene (**123**) [109], 3β,7α-dihydroxy-24-methyl-cholest-5,22-diene (**124**) [110], 3β,7α-dihydroxy-cholest-5-ene (**125**) [110,111,112], cholest-4-ene-3-one (**126**) [113,114,115], 24- methylene-cholest-4-ene-3-one (**127**) [116,117], (22*E*)-cholest-4,22- dien-3-one (**128**) [116], and (22*E*)-24-methyl-cholest-4,22-dien-3-one (**129**) [118] (Figure 21), were isolated from the soft coral *Dendronephthya* sp. collected off coral reef in Sanya, Hainan Province, South China Sea of People’s Republic of China [119]. The structures of steroids **115**–**129** were elucidated by spectroscopic methods and by comparison of their spectroscopic data with those reported previously. However, the configuration of Me-28 at stereogenic center C-24 in steroids **116**, **119**, **124**, and **129** were not determined in this study. Steroids **115**, **116**, and **120** belonging to 3-*O*-formylated cholesterol analogues and steroids **117**–**119** are unique ring A-contracted steroids [119].

**Figure 22:**
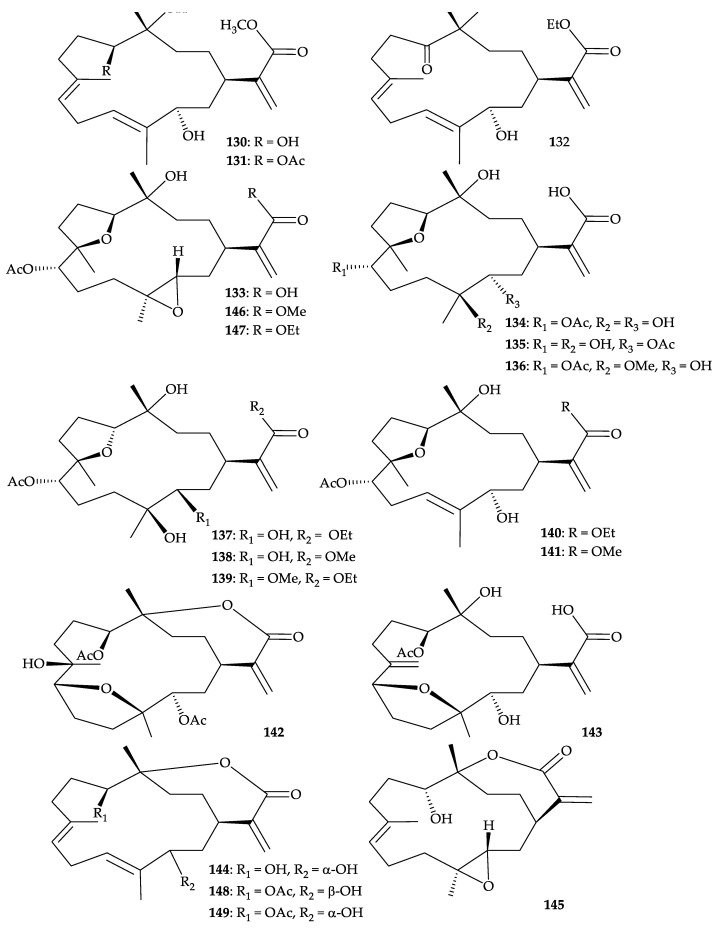
Structures of dendronpholides C–F (**130**–**133**), I–R (**134**–**143**), (−)-sandensolide (**144**), 11-episinulariolide (**145**) and sinulaflexiolides E, F, J, K (**146**–**149**).

A chemical examination of a soft coral identified as *Dendronephthya* sp., collected from the inner coral reef in Sanya Bay, Hainan Island of China, resulted in the isolation of 20 cembrane-type diterpenoids [120], including 15 new metabolites, dendronpholides C–F (**130**–**133**), I–R (**134**–**143**), and (–)-sandensolide (**144**) (an enantiomer of sandensolide) [120,121,122,123,124], along with five known compounds, 11-episinulariolide (**145**) [125,126,127,128,129,130], and sinulaflexiolides E, F, J, K (**146**–**149**) [128] (Figure 22). The structures of all isolates **130**–**149** were determined through spectroscopic methods and by comparison with those reported in literature [120]. Cembranoid dendronpholides C (**130**), J (**135**), and sinulaflexiolide E (**146**) showed cytotoxicity toward BGC-823 (human papillomavirus-related endocervical adenocarcinoma) cells with IC_50_ values of 0.05, 0.20, 0.02 µg/mL, respectively, whereas the other compounds were not active. A comparison of the cytotoxic data between **130** and **144** revealed that the methyl ester functionality plays a crucial role in the inhibition of BGC-823 cells compared to the the ε-lactone functionality. This is the first report of cembrane-type diterpenoids from the soft corals belonging to the genus *Dendronephthya* [120].

**Figure 23:**
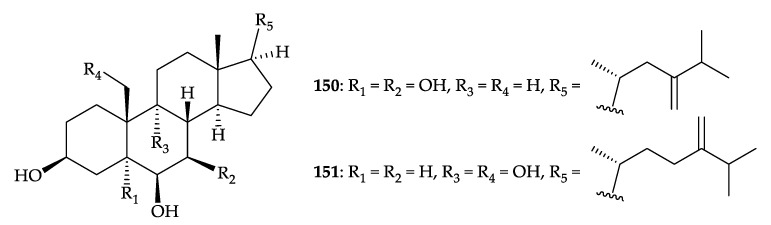
Structures of 23-nor-ergost-24-ene-3β,5α,6β,7β-tetrol (**150**) and ergost-24-ene-3β, 6β,9α,19-tetrol (**151**).

In 2010, two tetrahydroxylated sterols, including a new compound, 23-nor-ergost-24-ene- 3β,5α,6β,7β-tetrol (**150**) and a known analogue, ergost-24-ene-3β,6β,9α,19β-tetrol (**151**) [131], were isolated from *Dendronephthya* sp. collected from Naozhou Islands of the South China Sea [132] (Figure 23). The structures of sterols **150** and **151** were identified by spectroscopic methods [132]. Sterol **150** showed cytotoxicity toward the BEL-7402, MCG (human plasma cell myeloma), MCF, LoVo (human colorectal adenocarcinoma), and Hep G2 (human hepatocellular carcinoma) cells with IC_50_ values of 32.2, 20.5, 2.0, 5.5, and 18.6 µg/mL, respectively, and sterol **151** was cytotoxic against MCG and LoVo cells (IC_50_ = 22.0, 13.8 µg/mL), respectively [132].

**Figure 24:**
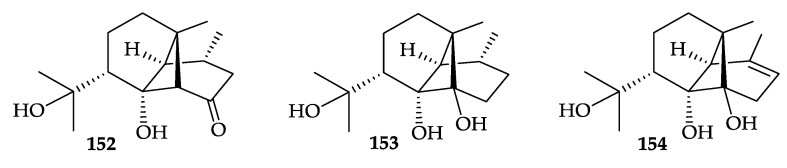
Structures of dendronephthols A–C (**152**–**154**).

Three new ylangene-type sesquiterpenoids, dendronephthols A–C (**152**–**154**) (Figure 24), together with two known steroids, dendronesterone A (**10**) [8] (Figure 3) and cholesterol (**26**) [30] (Figure 5), were isolated from a Red Sea soft coral *Dendronephthya* sp., collected near the coast of Hurghada, Egypt [133]. The structures of new sesquiterpenoids **152**–**154** were established by spectroscopic methods and **152** and **154** were found to be cytotoxic against L5178Y (mouse lymphoma) cells with ED_50_ values of 8.4 and 6.8 µg/mL, respectively [133].

**Figure 25:**
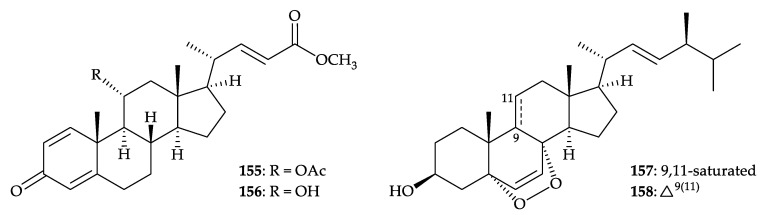
Structures of dendronesterones D (**155**) and E (**156**), 5α,8α-epidioxy-24(*S*)-methyl- cholesta-6,22-dien-3β-ol (**157**), and 5α,8α-epidioxy-24(*S*)-methylcholesta-6,9,22-trien-3β-ol (**158**).

Furthermore, two new steroids, dendronesterones D (**155**) and E (**156**), featuring with 1,4-dienone moiety, together with three known steroids, methyl 3-oxochola-4,22-dien-24-oate (**110**) [105] (Figure 19), 5α,8α-epidioxy-24(*S*)-methylcholesta-6,22-dien-3β-ol (**157**), and 5α,8α-epidioxy- 24(*S*)-methylcholesta-6,9,22-trien-3β-ol (**158**) [36,134], were isolated from an octocoral *Dendronephthya* sp., collected off the northeast coast of Taiwan [135] (Figure 25). The structures of new steroids **155** and **156** were elucidated by using spectroscopic methods and **155** was found to suppress the expression of inducible nitric oxide synthase (iNOS) and cyclooxygenase-2 (COX-2) to 24.2 and 70.4% at a concentration of 10 µM [135].

**Figure 26:**
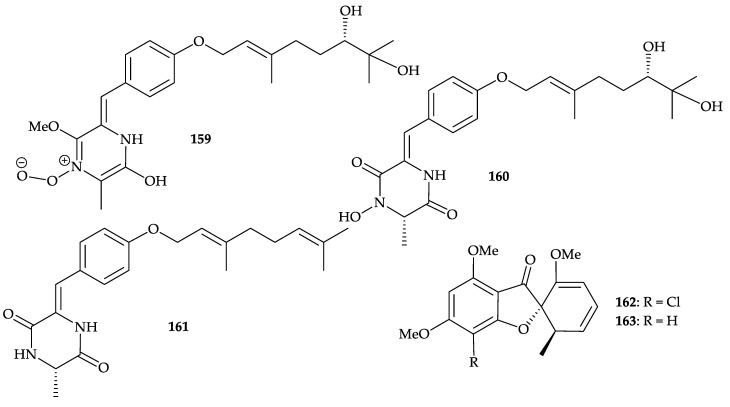
Structures of janthinolides A (**159**), B (**160**), deoxymycelianamide (**161**), griseofulvin (**162**), and dechlorogriseofulvin (**163**).

Two new 2,5-piperazinedione derivatives, janthinolides A (**159**) and B (**160**), as well as a new natural product, deoxymycelianamide (**161**) [136,137], and two known metabolites, griseofulvin (**162**) [138,139,140,141,142], and dechlorogriseofulvin (**163**) [142,143,144], were isolated from the fermentation broths of the endophytic fungus *Penicillium janthinellum*, isolated from a soft coral identified as *Dendronephthya* sp., collected in the South China Sea [145]. The structures of metabolites **159**–**163** were determined by spectroscopic data analysis and compound **162** displayed inhibitory concentration at 2.75 and 20 µg/mL against the fungal pathogen *Alternaria solani* and ascomycetous pathogen *Pyricularia oryzae*, respectively [145] (Figure 26).

**Figure 27:**
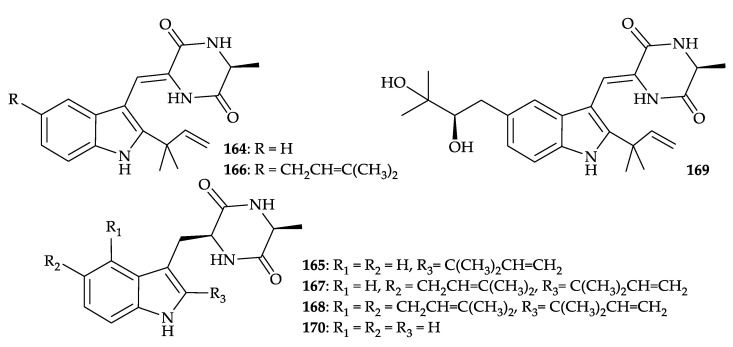
Structures of neoechinulin A (**164**), preechinulin (**165**), isoechinulin A (**166**), tardioxopiperazine A (**167**), variecolorin L (**168**), dihydroxyisoechinulin A (**169**), and L-alanyl-L- tryptophan anhydride (**170**).

Moreover, seven isoechinulin-type alkaloids, neoechinulin A (**164**) [146,147,148,149,150,151,152,153,154], preechinulin (**165**) [155,156], isoechinulin A (**166**) [149,157], tardioxopiperazine A (**167**) [158], variecolorin L (**168**) [159], dihydroxyisoechinulin A (**169**) [160], and L-alanyl-L-tryptophan anhydride (**170**) [161] (Figure 27), were isolated from the fermentation broths of an endophytic fungus *Nigrospora oryzae* isolated from a soft coral identified as *Dendronephthya* sp. collected in the South China Sea [162]. The structures of **164**–**170** were determined by their spectroscopic data and by comparison with those reported in the literature. In the antifouling activity against the larval settlement of barnacle *Balanus amphitrite*, compound **166** showed activity with an IC_50_ value of 5.92 µg/mL [162].

## 11. Conclusions

**Figure 28:**
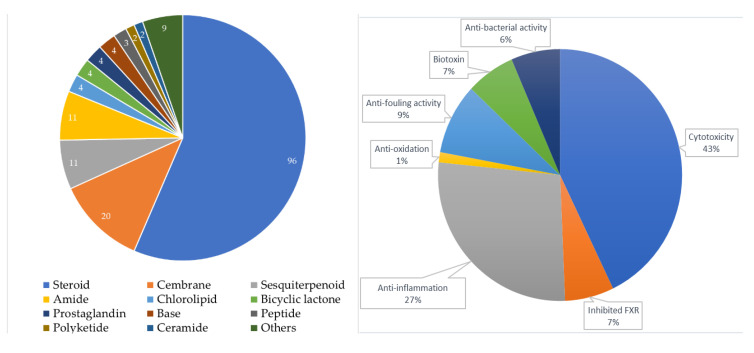
Biomedical activities of natural products from *Dendronephthya* spp.

Ever since the *seco*-steroids, isogosterones A–D (**1**–**4**) were obtained from a specimen of the octocoral *Dendronephthya* collected off the Izu Peninsula, Japan [2], 170 interesting secondary metabolites, including 96 steroids (56.47%), 20 cembranes (11.76%), 11 sesquiterpenoids (6.47%), 11 amides (6.47%), 4 chlorolipids (2.35%), 4 bicyclic lactones (2.35%), 4 prostaglandins (2.35%), 4 bases (2.35%), 3 peptides (1.76%), 2 polyketides (1.18%), 2 ceramides (1.18%), 1 glycerol (0.59%), 1 glycyrrhetyl amino acid (0.59%), 1 benzoic acid (0.59%), 1 trigonelline (0.59%), 1 hexitol (0.59%), 1 pentanoic acid (0.59%), 1 octadecanone (0.59%), 1 aminoethyl ester (0.59%), and a 53-KD allergenic component (0.59%), were produced by *Dendronephthya* spp., and extensive biomedical activities, especially in cytotoxicity and anti-inflammatory activity, were related to these natural substances (Figure 28).

All the secondary metabolites from *Dendronephthya* spp., reported between 1999 and 2019 were obtained from the octocorals distributed in the Indo-Pacific Ocean and Red Sea. As more than 56% of the compounds obtained from the *Dendronephthya* genus are steroids, based on above findings, these results suggest that continuing the investigation of new steroid analogues with the potential bioactivities from this marine organism are worthwhile for further development. The octocoral *Dendronephthya* sp. had been transplanted to culturing tanks located in the National Museum of Marine Biology and Aquarium, Taiwan, for the extraction of additional natural products to establish a stable supply of bioactive material.
